# Co-Administration of the Traditional Medicines Hachimi-Jio-Gan and Hochu-Ekki-To Can Reverse Busulfan-Induced Aspermatogenesis

**DOI:** 10.3390/ijms21051716

**Published:** 2020-03-03

**Authors:** Ning Qu, Miyuki Kuramasu, Kenta Nagahori, Yuki Ogawa, Shogo Hayashi, Yoshie Hirayanagi, Hayato Terayama, Kaori Suyama, Kou Sakabe, Masahiro Itoh

**Affiliations:** 1 Department of Anatomy, Division of Basic Medical Science, Tokai University School of Medicine, Kanagawa 259-1193, Japan; terahaya@tokai-u.jp (H.T.);; 2 Department of Anatomy, Tokyo Medical University, Tokyo 160-8402, Japan; kitaoka@tokyo-med.ac.jp (M.K.); kenta-n@tokyo-med.ac.jp (K.N.); yogawa@tokyo-med.ac.jp (Y.O.); hirayanagi@ndmc.ac.jp (Y.H.); itomasa@tokyo-med.ac.jp (M.I.); 3 Department of Anatomy, School of Medicine, International University of Health and Welfare, Chiba 286-8686, Japan; sho5-884@umin.ac.jp

**Keywords:** anti-cancer treatment, aspermatogenesis, testicular immunology, oriental medicine, co-administration

## Abstract

Busulfan is used as a chemotherapeutic drug to treat childhood and adult chronic myelogenous leukemia, and as an immunosuppressive agent before bone marrow transplantation. A key side effect of busulfan is the alteration of male reproductive function. Infertility caused by anti-cancer treatments has become a significant concern, but there are currently limited treatments for this condition. Recently, we demonstrated that Gosha-jinki-gan, a traditional Japanese medicine, completely reversed the spermatogenesis defects caused by cancer treatment in mice. Hochu-ekki-to and Hachimi-jio-gan are commonly used to treat male infertility, and Hachimi-jio-gan shares herbal ingredients with Gosha-jinki-gan. Therefore, in the present study, we administered Hachimi-jio-gan and Hochu-ekki-to alone or in combination to mice with severe aspermatogenesis caused by busulfan treatment. We performed testis weight measurements, quantitative histological assessments of the testes and the epididymis, and evaluated sperm counts and morphology. We also assessed the expression of immune mediators and macrophage markers. Treatment with a combination of both the medicines significantly reduced busulfan-induced testicular toxicity when compared to the lone treatment with either medicine. We demonstrated that treatment efficacy was related to a differential impact on testicular inflammation, and that the synergistic effect of co-administration completely reversed the busulfan-induced damage to the reproductive functions.

## 1. Introduction

Male infertility and abnormalities in sperm production can be caused by a variety of disorders, including anatomical problems, hormonal imbalances, genetic defects, and psychological problems or behavioral problems [1]. Male sterility is a frequent side effect after cancer treatment, as alkylating agents and irradiation evoke testicular damage that results in prolonged azoospermia. However, there is little information on the treatments for male infertility after cancer therapy.

Oriental herbal medicines have been reported to improve total sperm count and motility. These include the Hachimi-jio-gan and Hochu-ekki-to Japanese traditional medicines, which are clinically used to treat male infertility. Hachimi-jio-gan, which is also called BaWei DiHuang Wan in Chinese, is a herbal mixture containing eight ingredients (Table 1). It was discovered more than 1800 years ago by the notable Dr. Zhongjing Zhang [2] and was used in traditional medicine to treat diabetes and urinary disorders [3,4]. Hachimi-jio-gan increases serum 17β-estradiol levels and improves spermatogenesis in oligozoospermic men [5]. Hochu-ekki-to, also known as BuZhong YiQi Wan in Chinese, is a herbal mixture containing ten ingredients (Table 1) and is clinically used to treat idiopathic male infertility. Ishikawa et al. investigated the changes in the responsiveness to human chorionic gonadotropin (hCG) stimulation in 63 infertile men and demonstrated that Hochu-ekki-to corrected the Leydig cell dysfunctions in some infertile men, resulting in improvements in sperm quality [6]. Additionally, Nakayama et al. demonstrated that Hochu-ekki-to was able to promote the synthesis of proteins involved in the functional maturation of spermatozoa in the epididymis [7]. Furthermore, Hochu-ekki-to was demonstrated to enhance sperm quality by improving semen conditions, including reducing seminal plasma *IL-6*, which inversely correlated with sperm concentration [8,9,10]. Moreover, treatment with Hochu-ekki-to before an influenza infection in mice significantly increased the expression of interferon-alpha (*IFN-α*), several Toll-like receptors (*TLRs*), and defensins [11,12].

Busulfan (BSF; 1,4-butanediol methanesulfonate) is a chemotherapeutic agent that is used to treat various malignancies [13,14], and is also commonly used prior to hematopoietic stem cell transplantation [13]. Some studies have shown that BSF exposure in mice significantly decreased concentrations of deacetylated *p53*, resulting in spermatogonial cell resistance to apoptosis [15]. BSF exposure has also been shown to up-regulate the tumor necrosis factor-alpha (*TNF-α*) and macrophage chemotactic protein 1 (*MCP-1*) expression in the Sertoli cells, and to facilitate macrophage infiltration into the testes [16]. Additionally, damaged germ cells in the BSF-treated mice release endogenous *TLR* ligands to activate *TLR2* and *TLR4* in the Sertoli cells, thus initiating endogenous inflammation in the testes [16,17]. Recently, we have found that Gosha-jinki-gan, an oriental Japanese medicine, was able to completely normalize testicular immunopathology and promote the recovery from severe aspermatogenesis after BSF treatment in mice [17]. As both Hochu-ekki-to and Hachimi-jio-gan have been used to treat male infertility, and Hachimi-jio-gan consists of the same herbal ingredients found in Gosha-jinki-gan except for *Achyranthis Radix* and *Plantaginis Semen* (Table 1), we hypothesized that these traditional medicines may also cure testicular damage after BSF treatment. To our knowledge, there are no clinical reports on the effects of either Hachimi-jio-gan or Hochu-ekki-to on chemotherapy-induced infertility. In this study, we compared the effects of Hachimi-jio-gan and Hochu-ekki-to, administered alone or in combination, on BSF-induced infertility. We further examined the immunological factors that are involved in spermatogenesis after anti-cancer treatment.

## 2. Results

### 2.1. The Damaging Effects of Busulfan-Treatment in Mice on Day 60

**Figure 1:**
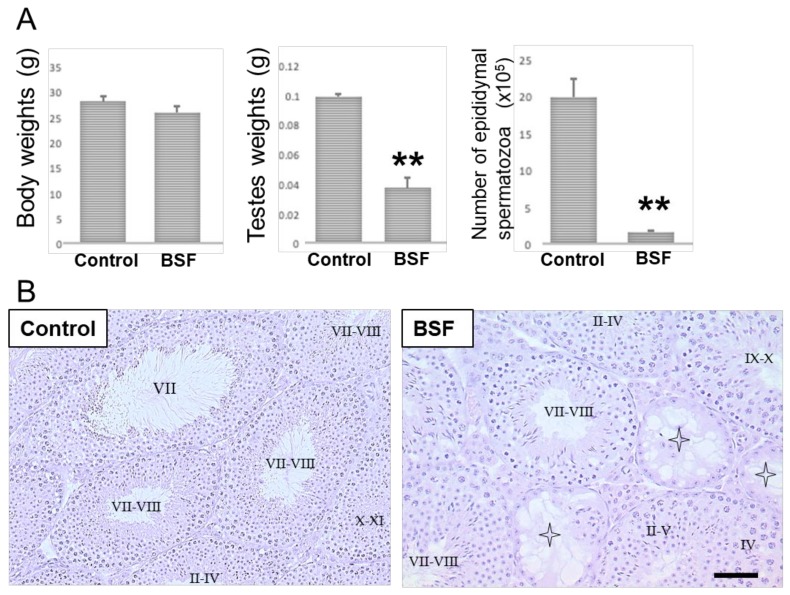
Body weights and reproductive changes in the DMSO-treated (control, *n* = 5) and the busulfan-treated (BSF, *n* = 5) mice on day 60 after a single intraperitoneal administration. (**A**) Body weights, testis weights, and the epididymal spermatozoa counts have been presented as mean ± standard deviation. ** *p* < 0.01 vs. the control group. (**B**) Light microscopic analyses. Sections stained with hematoxylin and eosin showing seminiferous tubules in testes (representative images from *n* = 5 mice per group). Intact seminiferous tubules showing normal stages (I-XII) of spermatogenesis from spermatogonia to spermatozoa in the cycle of germinal epithelium were observed in the control group testes. Atrophic seminiferous tubules (star pattern) with azoospermia were observed in the BSF group. Scale bar = 50 µm.

There were no significant differences in the body weights of the BSF-treated mice compared to mice in the control group (which were treated with dimethyl sulfoxide) on day 60 after a single intraperitoneal administration of the drugs (Figure 1A). However, the testicular weights and the epididymal spermatozoa counts (the number of spermatozoa with a short, thick, and sickle-shaped head) were significantly lower in the BSF-treated mice compared to the control mice (Figure 1A). In the control mice, spermatogenesis, showing different stages of spermatogenesis (stage II–IV, VII, VII–VIII or X–XI) from spermatogonia to spermatozoa in the cycle of germinal epithelium, were observed in the seminiferous tubules (Figure 1B). On the other hand, in the BSF-treated mice, the presence of both atrophic seminiferous tubules (63.51 ± 3.27 per 100 seminiferous tubules) and intact seminiferous tubules with spermatogenesis (stages of spermatogenesis II–IV, II–V, IV, VII–VIII, or IX–X) was observed. It was also noted that almost all spermatocytes and spermatids had disappeared in these atrophic seminiferous tubules (Figure 1B).

### 2.2. Body Weight, Testis Weight, and Sperm Count in Mice of Each Group on Day 120

**Figure 2:**
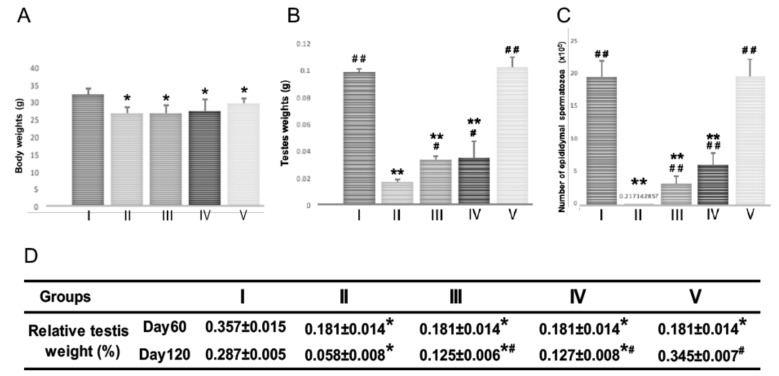
Body weight, testis weight, and sperm count after BSF treatment followed by diet supplementation with Hachimi-jio-gan and/or Hochu-ekki-to. (**A**) Body weight, (**B**) Testis weight, and (**C**) Epididymal spermatozoa count of mice in each group at Day 120. (**D**) Relative testis weight in each group at Day 60 and Day 120. Relative testis weight was calculated in percentage by dividing the combined weight of both testes in milligrams by body weight in grams. Data have been presented as mean ± standard deviation. * *p* < 0.05 and ** *p* < 0.01 vs. the control group; ^#^ *p* < 0.05 and ^##^ *p* < 0.01 vs. the BSF group.

After BSF treatment at the start of the experiment (day 0), the mice were fed with a standard diet for 60 days before some were switched to the diets supplemented with both Hachimi-jio-gan (TJ7) and Hochu-ekki-to (T41), or with either medicine alone, for a further 60 days. At the end of the experiment (day 120), the mean body weights of the mice in all the BSF-treated groups were significantly lower than mice in the control group, regardless of TJ7 or T41 supplementation (Figure 2A). In contrast, the BSF-treated mice that received both TJ7 and T41 showed significant and almost complete recoveries in their testicular weights and epididymal spermatozoa counts, to levels that were comparable to the mice that did not receive BSF treatment (Figure 2B–D). The BSF-treated mice that received TJ7 or T41 alone also showed significant recoveries in their testicular weights and spermatozoa counts compared to the BSF-treated mice that did not receive diet supplementation. However, recoveries due to the single administration of either drug were not as marked as the recoveries due to the administration of both the drugs in combination, and the parameters measured did not return to the control levels (Figure 2B–D).

### 2.3. Analyses of Testicular Spermatogenesis and Epididymal Spermatozoa in Mice on Day 120

**Figure 3:**
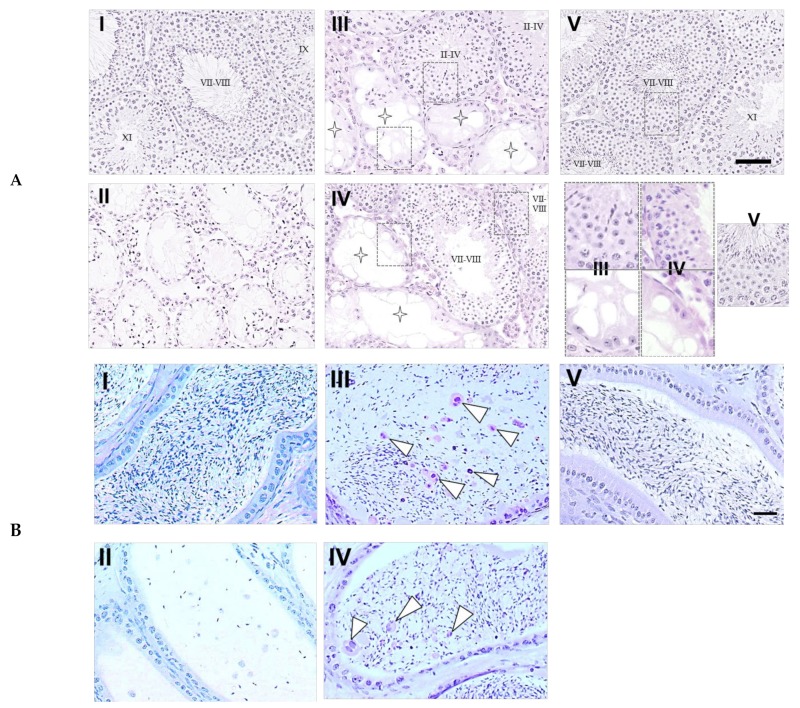
Testicular and epididymal sections from mice after BSF treatment followed by diet supplementation with Hachimi-jio-gan and/or Hochu-ekki-to. (**A**) Intact seminiferous tubules exhibiting all the stages of maturation of the germinal epithelium from spermatogonia to spermatozoa have been shown in the control group (I). Atrophic seminiferous tubules with azoospermia have been shown in the BSF group (II). Atrophic seminiferous tubules with aspermatogenesis (star pattern) and intact seminiferous tubules with spermatogenesis were observed in the BSF-treated mice administered with TJ7 (III) or TJ41 (IV) alone. Seminiferous tubules of normal appearance were observed in the BSF-treated mice co-administered with TJ7 and TJ41 (V). Enlarge part of seminiferous tubules bounded by dotted frames showed germinal epithelium of normal spermatognesis or aspermatogenesis. Scale bar = 50 µm. (**B**) Epididymal spermatozoa in the epididymal tubules of cauda epididymidis. The arrow heads indicate abnormal form of sperm. Spermatozoa with normal morphology appeared in the epididymal tubule in the control group (I). Only very few spermatozoa (and these did not have tails) were observed in the epididymal tubules in the BSF-treated mice (II). A loss of sperm density with some abnormal forms of spermatozoa (arrow heads) was observed in the BSF-treated mice administered with TJ7 (III) or TJ41 (IV) alone. Spermatozoa exhibited normal morphology and were present at high densities in the epididymal tubule in the BSF-treated mice co-administered with TJ7 and TJ41 (V). Scale bar = 50 µm.

Upon histological examination of the testes (Figure 3A), the intact seminiferous tubules showing the normal stages of spermatogenesis, from spermatogonia to spermatozoa (stages of spermatogenesis VII–VIII, IX, and XI) in the cycle of germinal epithelium, were observed in the control mice that did not receive BSF treatment. The BSF treatment induced the appearance of atrophic seminiferous tubules (91.65 ± 5.31 per 100 seminiferous tubules) and azoospermia in mice that did not receive diet supplementation. In mice that received TJ7 or TJ41 alone, the partial recovery of spermatogenesis was observed. Furthermore, in these mice, the seminiferous tubules that exhibited intact spermatogenesis (stages of spermatogenesis II–IV or VII–VIII) were found adjacent to the seminiferous tubules that showed aspermatogenesis as Sertoli cells only (enlarge part bounded by dotted frames in Figure 3A). In contrast, the co-administration of both TJ7 and TJ41 restored normal spermatogenesis in all the seminiferous tubules (stages of spermatogenesis VII–VIII).

The epididymal spermatozoa were also analyzed (Figure 3B), and mature spermatozoa with normal morphology were observed in the epididymal tubules of cauda epididymidis in the control mice. In the BSF-treated mice, only very few spermatozoa (and these did not have tails) were detected in the epididymal tubules of cauda epididymidis. In mice that received TJ7 or TJ41 alone, the spermatozoa were sparse and some abnormal spermatozoa were detected, but the pathology was less severe compared with that in the BSF-treated mice that did not receive either drug. The co-administration of both TJ7 and TJ41 restored normal epididymal morphology, and the spermatozoa densities were returned to the high levels observed in the control mice.

### 2.4. Proliferation and Apoptosis in the Testes of Mice

**Figure 4:**
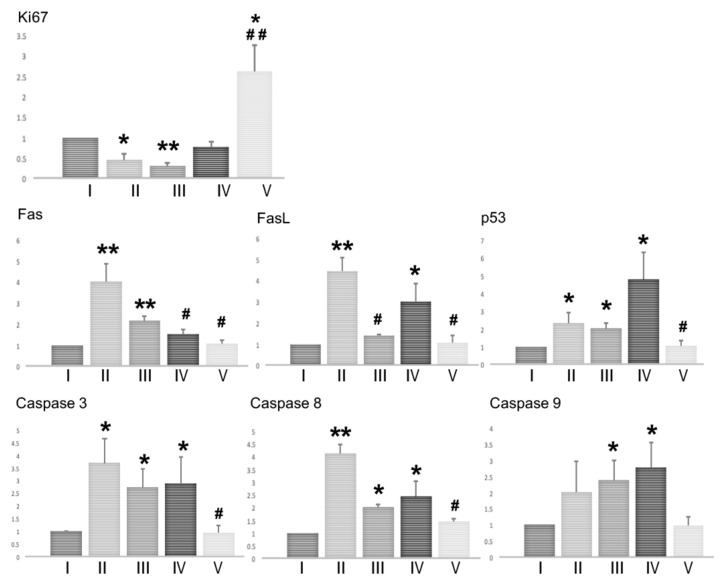
Effects of diet supplementation with Hachimi-jio-gan and/or Hochu-ekki-to on testicular proliferation and apoptosis. Expression of genes involved in proliferation (*Ki67*) and apoptosis (*Fas*, *FasL*, *Caspase3, Caspase8, Caspase9,* and *p53*) in testicular tissues of mice from each group on day 120. Data represent the mean values ± standard deviation of three mice in independent groups. * *p* < 0.05 and ** *p* < 0.01 vs. the control group; ^#^ *p* < 0.05 and ^##^ *p* < 0.01 vs. the BSF group.

We assessed the proliferation and apoptosis in the testes of mice at the end of the experiment (on day 120) by measuring the expression of genes involved in proliferation (*Ki67*) and apoptosis (*Fas*, *FasL*, *Caspase3, Caspase8, Caspase9,* and *p53*) in the testicular tissues (Figure 4). Testicular *Ki67* expression was significantly decreased in the BSF-treated mice, and in the BSF-treated mice that received TJ7, when compared with the mice in the control group. Supplementation with TJ41 alone restored *Ki67* expression to control levels, whereas supplementation with both TJ7 and TJ41 increased *Ki67* expression to levels that were significantly higher than that observed in the BSF group, and also in the control group.

BSF-treatment led to the increased expression of *Fas*, *FasL*, *Caspase3, Caspase8,* and *p53,* but not of *Caspase9.* Only the group that received both TJ7 and TJ41 effectively reduced the expression of all the apoptosis factors to levels seen in the control group. Supplementation with TJ41 alone decreased *Fas* expression but increased the *FasL* expression, whereas supplementation with TJ7 alone increased *FasL* expression but increased the *Fas* expression, when compared to the BSF-treated mice that did not receive diet supplementation. However, the administration of TJ7 or TJ41 alone increased the expression of *p53*, *Caspase3, Caspase8,* and *Caspase9* in the testes.

### 2.5. Expression of Immune Mediators and Macrophage Markers in the Testes

**Figure 5:**
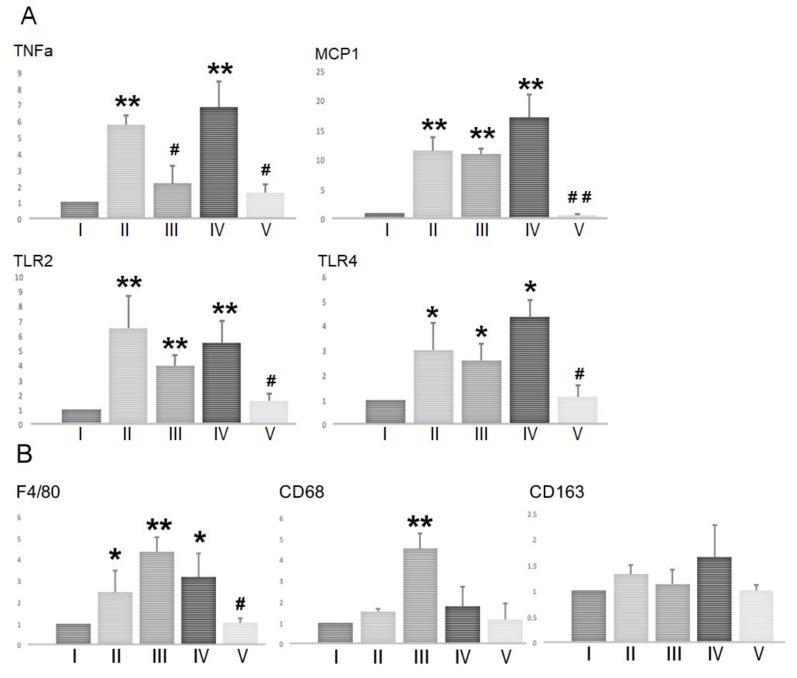
mRNA expression of (**A**) immune mediators and (**B**) macrophage markers in the testes after BSF treatment followed by diet supplementation with Hachimi-jio-gan and/or Hochu-ekki-to. Data represent the mean values ± standard deviation of three mice in independent groups. * *p* < 0.05 and ** *p* < 0.01 vs. the control group; ^#^ *p* < 0.05 and ^##^ *p* < 0.01 vs. the BSF group.

BSF treatment has been shown to induce spermatogenic cell damage by up-regulating *TNF-α* and *MCP-1* expression in the Sertoli cells via the activation of *TLR2* and *TLR4* [16]. Therefore, we examined the mRNA expression levels of these immune mediators in the testes (Figure 5A). BSF-treatment led to significant increases in the expression of *TNF-α, MCP-1, TLR2,* and *TLR4* compared to the controls. Supplementation with both TJ7 and TJ41 significantly reduced the BSF-induced increases in all four genes, which returned to control levels. In contrast, these increases were not alleviated by TJ41 alone. Furthermore, TJ7 alone was unable to reduce the elevated expression of *TNF-α*, but not *MCP-1, TLR2*, or *TLR4.*

Given that *MCP-1* facilitates macrophage recruitment, we also examined the effect of TJ7 and/or TJ41 on the expression of macrophage markers (at the mRNA level) in the testes (Figure 5B). The expression *F4/80* (a marker of total macrophages), *CD68* (a marker of newly-recruited macrophages), and *CD163* (a marker of resident macrophages) were analyzed. BSF treatment increased the expression of *F4/80*, but not the expressions of *CD68* and *CD163* compared to that of the controls. This increase in the *F4/80* expression was significantly reversed by the co-administration of both TJ7 and TJ41. Interestingly, *CD68* expression was significantly increased by TJ7 administration compared to the expression in all other groups.

## 3. Discussion

We examined whether the administration of Hachimi-jio-gan (TJ7) and Hochu-ekki-to (TJ41) alone or in combination could be effective for treating mice with severe aspermatogenesis caused by busulfan treatment. To our knowledge, this is the first study to demonstrate that the co-administration of the oriental medicines TJ7 and TJ41 completely reversed male infertility after busulfan chemotherapy.

The mice treated with BSF showed significant decreases in body weight, absolute and relative testes weights, and in their number of both proliferating spermatogonia and spermatozoa. This aspermatogenesis was irreversible unless the medication was administered [17]. Only the mice that subsequently received both TJ7 and TJ41 showed a complete recovery in their absolute and relative testes weights, the testicular proliferation, and in the numbers of epididymal spermatozoa (in both cell-counts and morphology) (Figure 2; Figure 3; Figure 4). However, no recoveries in their body weights were observed. The single administration of TJ7 or TJ41 alone led to the improvements in some reproductive parameters (such as testes weights and epididymal spermatozoa count), but these remained significantly compromised compared to that of the control mice and in the mice that received both TJ7 and TJ41 (Figure 2). These results indicated that only the co-administration of both TJ7 and TJ41 was able to completely regenerate the seminiferous epithelium that had been injured by BSF treatment.

A previous study demonstrated that BSF-induced damage up-regulated *TNF-α* and *MCP-1* expressions in the Sertoli cells via the activation of *TLR2* and *TLR4*, and induced extensive apoptosis in the germ cells [16,18,19,20,21,22]. Consistent with this previous study, we demonstrated that BSF treatment up-regulated the expression of *TNF-α, MCP-1, TLR2*, and *TLR4* in the testes. The up-regulation of these four genes was completely reversed by TJ7 and TJ41 co-administration (Figure 5), but was unaffected by TJ41 administration alone. Although *TNF-α* was inhibited by the administration of TJ7, the increased *MCP-1* induced an increase in mRNA of *F4/80* and *CD68* in the testes, and this can explain the fact that the remaining initiated endogenous inflammation in the testes. Furthermore, the activation of *TLR2* and *TLR4* by a TJ7 single administration triggered a common signaling pathway to up-regulate *Caspase8* and *Caspase3*-dependent apoptosis (Figure 4, Figure 5). These results are in agreement with the results reported by previous studies [19,20]. Therefore, although the single administration of TJ7 or TJ41 could not reverse the aspermatogenesis induced by BSF treatment, the co-administration of TJ7 and TJ41 surprisingly inhibited increases in damaged Sertoli cell factor expressions and significant apoptosis of germ cells in the testes after BSF treatment (Figure 4, Figure 5). These results are in line with our previous study on the effects of TJ107 on the BSF-induced aspermatogenesis [17]. Together, these discoveries demonstrated that traditional Japanese medicines may be able to reverse aspermatogenesis, and likely achieved this by reducing the BSF-induced immune mediator expression and the germ cell apoptosis in the testes. These findings verify the BSF-induced initiating endogenous inflammation in the testes and are worthwhile to provide novel insights into the mechanisms underlying the differences of single or combined administration in traditional medicine.

Over the last 20 years, fertility preservation after cancer treatment has become an emerging discipline, owing to an increased awareness among researchers, clinicians, and patients, as well as the extended survival of patients after cancer treatment [23]. However, there have been few effective clinical therapies for male infertility after chemotherapy [24]. Traditional Chinese and Japanese medicines have been widely applied to treat infertility due to their efficacy and minimal side effects. The traditional medicine theory encompasses Yin-Yo, a philosophical concept that contextualizes all matters from two opposite but complementary aspects in nature. This encompasses concepts of ‘Yin-Yo’ and ‘male-female,’ or Yin-Yang in Chinese; Kyo (deficiency)-Jitsu (excess) (or Xu-Shi in Chinese); and Ki (vital energy)-Ketsu (blood)-Sui (fluid) (or Qi-Xue-Shui in Chinese) [25]. On the basis of the oriental theory, the term “kidney (Jin-Ki in Japanese)” not only refers to the kidney, but also considers human growth, development, and reproduction [26]. Therefore, the concept of “kidney replenishment” is important and related to spermatogenesis. Consistent with this concept, we have demonstrated that Gosha-jinki-gan (TJ107), a “kidney-replenishing” herbal medicine, was effective in reversing infertility after cancer treatment [17]. The herbal medicines *Plantaginis Semen* and *Achyranthis Radix*, which are present in TJ107 but not in TJ7, have been hypothesized to replenish Yo deficiency (based on the Yin-Yo and the Kyo-Jitsu theories), and may contribute to the rectification of the kidney deficiencies. TJ7, which is commonly used in Yin-pattern patients, is a well-known traditional medicinal formula for treating idiopathic male infertility [27] and oligozoospermia [28], and is effective at improving male fertility. In contrast, TJ41, commonly used in Yo-pattern patients, is frequently used to improve both immunosuppression and deficiencies of Ki, Ketsu, and Sui in cancer patients [29]. Furthermore, Satoh et al. found that TJ41 increased lymphocyte cell-surface antigens, the CD3-positive cells, and the CD3/CD4 double-positive cells in elderly patients with chronic weakness, and these increased immune functions were associated with an increased quality of life [30]. 

Although traditional Chinese and Japanese medicines have been widely used to treat infertility due to their efficacy and minimal side effects, studies that demonstrate their pharmacological effects and mechanisms of action are limited. We were able to demonstrate the pharmacological effects of TJ7 and TJ41 on infertility after cancer treatment in this study. Our data demonstrated that the administration TJ7 or TJ41 alone was unable to effectively reduce testicular immune-related inflammation induced by BSF and did lead to the recovery of spermatogenesis. The combined administration of both TJ7 and TJ41 effectively restored reproductive function after BSF treatment, recapitulating the effect of TJ107, which was to replenish Yo deficiency during the rectification of kidney deficiencies. We also suggest that these medicines may act by reducing the testicular inflammation induced by BSF. We have previously demonstrated that impaired reproductive function induced by chemotherapy and radiotherapy was related to immune-pathophysiologies [17,31,32]. In the future, we propose to examine the effects of TJ7 and TJ41 alone or in combination on irradiation-induced spermatogenic defects and to further clarify their mechanisms of action, focusing on testicular immunology. These basic experiments will guide the discoveries of clinical therapies for male infertility after cancer treatment.

## 4. Materials and Methods

### 4.1. Animals

C57BL/6J male mice at 4 weeks of age (weighing 16–20 g) were purchased from SLC (Shizuoka, Japan) and were kept in the Laboratory Animal Center of Tokyo Medical University (Tokyo Medical University Animal Committee; No. S28016-01042016 and No. H290048-01042017) and the Animal Laboratory of Support Center for Medical Research and Education, Tokai University (Tokai University Animal Committee; No. 171058-01042017 and No. 181040-01042018). They were maintained at a temperature of 22–24 °C and at a relative humidity of 50%–60%, with a 12 h light–dark cycle.

### 4.2. Preparation of Diets Containing Oriental Medicines

The Hachimi-jio-gan (TJ7) (extract granules in a powdered form; No. 2120007010, 2130007010, 2150007010) and Hochu-ekki-to (TJ741) (extract granules in a powdered form; No. 2120041010, 21200041020, 21700041010) were manufactured by Tsumura & Co. (Tokyo, Japan) according to the Japanese and international manufacturing guidelines. The diets supplemented with oriental medicines were prepared as a standard mouse diet (standard MF diet; 23.1% crude protein [w/w], 5.1% crude fat, 5.8% crude ash, 2.8% crude fiber, and 55.3% nitrogen-free extract and mineral mixture) containing 4.8% [w/w] TJ7 (TJ7 diet), 6.0% TJ41 (TJ41 diet), 4.8% TJ7, and 6.0% TJ41 (TJ(7+41) diet) by Oriental Yeast Co., Ltd. (Tokyo, Japan).

### 4.3. Experimental Design

The BSF (Sigma, St. Louis, MO, USA) was first dissolved in dimethyl sulfoxide (DMSO; Sigma) and 100 μL of distilled water was added to achieve a final concentration of 40 mg/kg according to previously described methods [17,33].

**Figure 6:**
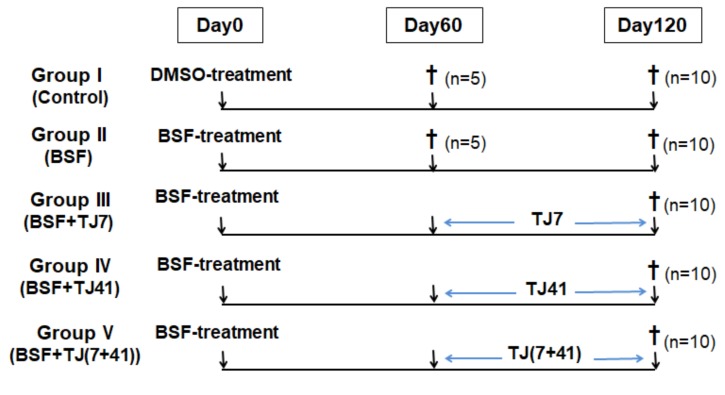
Time schedule of treatment for the mice in each group.

The mice were randomly divided into five groups. The BSF treatment was delivered as a single intraperitoneal (i.p.) injection on day 0, except to the mice in the control group, which received DMSO instead of BSF. All mice were fed with the standard MF diet for 60 days, and some groups of mice were switched to the different Japanese medicine-supplemented diets for a further 60 days (Figure 6). The five groups were as follows: Group I (Control; *n* = 15; DMSO sham treatment, and fed with the standard MF diet for 120 days); Group II (BSF; *n* = 15; BSF treatment, and fed with the standard MF diet for 120 days); Group III (BSF + TJ7; *n* = 10; BSF treatment, fed with the standard MF diet for 60 days, and switched to the TJ7 diet for the next 60 days); Group IV (BSF + TJ41; *n* = 10; BSF treatment, fed with the standard MF diet for 60 days, and switched to the TJ41 diet); Group V (BSF + TJ(7+41); *n* = 10; BSF treatment, fed with the standard MF diet for 60 days, then switched to the TJ7 + TJ41 diet.

The general condition, food intake, and body weight of each mouse was recorded at 10-day intervals from 60 days to 120 days after injection. All the experimental protocols in this study were carried out in accordance with the guidelines of the National Institutes of Health and were approved by the Tokyo Medical University Animal Committee (No. S28016 and No. H290048) and the Tokai University Animal Committee (No. 171058 and No. 181040). Sixty days after injection, five mice each from the control and the BSF groups were anesthetized with pentobarbital (65 mg/kg body weight) and their testes and epididymis were immediately removed. One hundred and twenty days after injection, the testes and epididymis from all remaining mice (*n* = 10 from each group) were removed using the same procedure. The testis weight was measured and the relative testis weight was calculated in percentage by dividing the combined weight of both testes in milligrams by body weight in grams. The data have been presented as mean ± standard deviation (SD).

### 4.4. Histological Examination of the Testes and Epididymis

The testes and epididymis of mice from the control and the BSF groups on day 60 (*n* = 5 per group) and from all the groups on day 120 (*n* = 5 per group) were fixed with the Bouin’s solution and embedded in plastic (Technovit7100; Kulzer & Co., Wehrheim, Germany). For histological examination, sections (5 μm thick) were cut at 15 μm–20 μm intervals and were stained with Gill hematoxylin and 2% eosin Y (Muto PC, Tokyo, Japan) for observation under light microscopy. In all testes, more than 200 round and oval cross sections of the seminiferous tubules in each testis were checked simultaneously by two senior histologists, who were unaware of the experimental group of each sample. The results are presented as the stages of spermatogenesis [34,35] and the percentage of atrophic seminiferous tubules of mean ± SD of number of tubular sections without the presence of elongating or elongated spermatids/100 tubular sections in each testis.

### 4.5. Analysis of the mRNA Expressions of Cytokines Using Real-Time RT-PCR

The total RNA was purified from fresh testes obtained at day 120 (*n* = 5 per group) using the TRIzol RNA Extraction Kit (Invitrogen, Carlsbad, CA) according to the manufacturer’s instructions, and the RNA pellets were dissolved in 10 mL of RNase-free distilled water. The total RNA quality and quantity was measured at 260/280 nm using a UV spectrophotometer and was stored at −80 °C prior to use. The cDNA was prepared by reverse transcription (RT) from 10 μg of total RNA in a 100 μL reaction mixture, using random primers according to a standard protocol (high-capacity cDNA archive kit; PE Applied Biosystems, Foster City, CA). The iCycler thermal cycler (Bio-Rad, Hercules, CA) was used to perform the PCR reactions, and the mixtures were stored at −80 °C until analysis. Real-time RT-PCR was performed using 3 ng of cDNA with the validated SYBR^(R)^ green gene expression assay in combination with SYBR Premix Ex Taq^TM^ (TaKaRa, Bio Inc., Ohtsu, Japan) for measuring *Ki67, Fas, FasL, Caspase3, Caspase8, Caspase9, p53, F4/80, CD168, CD63, TNF-α, MCP-1, TLR2, TLR4,* and *GAPDH* gene transcripts. Quantitative real-time PCR was performed in duplicate with the Thermal Cycler Dice Real-time System TP800 (TaKaRa). The Thermal Cycler Dice Real-time System software (TaKaRa) was used to analyze the data, and the comparative C_t_ method (2∆∆C_t_) was used to quantify the gene expression levels. The results were expressed relative to the amount of *GAPDH* transcript used as an internal control. Table 2 lists all the primers used in this analysis.

### 4.6. Epididymal Spermatozoa Count

To determine the number of spermatozoa, the epididymal spermatozoa were recovered from the epididymis of mice in the control and the BSF groups at day 60 (*n* = 5 per group) and from all the groups at day 120 (*n* = 5 per group). Briefly, the epididymis was excised and cut into six pieces in phosphate-buffered saline (PBS). The pieces were gently stirred with a pipette and were passed through a stainless-steel mesh. The spermatozoa were harvested by centrifugation at 400× *g* for 10 min and were resuspended in 5 mL of PBS after washing thrice with PBS. Normal mouse spermatozoon has a short, thick, and sickle-shaped head. All samples were checked simultaneously by two senior histologists, who were unaware of the experimental group of each sample.

### 4.7. Statistical Analysis

An ANOVA and Tukey–Kramer post-hoc test was used to analyze the differences between the multiple groups. A *p*-value < 0.05 was considered statistically significant.

## 5. Conclusions

**Figure 7:**
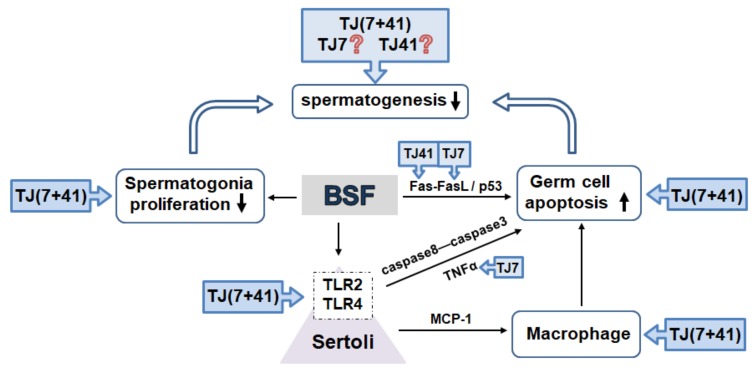
The traditional Japanese medicine modulated testicular immune-related inflammation. BSF-treatment disrupted spermatogenesis by decreased (↓) proliferation and increased (↑) apoptosis in the germ cells. Activated *TLR2* and *TLR4* expression in the Sertoli cells (bounded by dotted frames) induced extensive apoptosis in the germ cells.

This is the first study to examine whether the single or co-administration of the traditional medicines Hachimi-jio-gan and Hochu-ekki-to reverses the BSF-induced male infertility. BSF-induced damage up-regulated the *TNF-α* and *MCP-1* expressions in the Sertoli cells via the activation of *TLR2* and *TLR4*, and induced extensive apoptosis in the germ cells. Only the co-administration of both the medicines had a synergistic therapeutic effect and restored the spermatogenesis by modulating testicular immune-related inflammation (Figure 7).

## Figures and Tables

**Table 1:** Medicinal components of Hachimi-jio-gan, Hochu-ekki-to, and Gosha-jinki-gan (ratio).

Hachimi-jio-gan (TJ7)	Hochu-ekki-to (TJ41)	Gosha-jinki-gan (TJ107)
*Rehmanniae radix* (6.0 g)	*Astragali radix* (4.0 g)	*Rehmanniae radix* (5.0 g)
*Corni fructus* (3.0 g)	*Atractylodis lanceae rhizome* (4.0 g)	*Achyranthis radix* (3.0 g)
*Dioscoreae rhizoma* (3.0 g)	*Ginseng radix* (4.0 g)	*Corni fructus* (3.0 g)
*Alismatis rhizoma* (3.0 g)	*Angelicase radix* (3.0 g)	*Dioscoreae rhizome* (3.0 g)
*Hoelen* (3.0 g)	*Bupleuri radix* (2.0 g)	*Plantaginis semen* (3.0 g)
*Moutan cortex* (3.0 g)	*Zizyphi fructus* (2.0 g)	*Alismatis rhizome* (3.0 g)
*Cinnamoni cortex* (1.0 g)	*Aurantii nobilis pericarpium* (2.0 g)	*Hoelen* (3.0 g)
*Aconiti tuber* (0.5 g)	*Glycyrrhizae radix* (1.5 g)	*Moutan cortex* (3.0 g)
	*Cimicifugae rhizome* (1.0 g)	*Cinnamoni cortex* (1.0 g)
	*Zingiberis rhizome* (0.5 g)	*Aconite tuber* (1.0 g)

The standard dose of 7.5 g/day contained 4.0 g of TJ7, 5.0 g of TJ41, or 4.5 g of TJ107 in powdered form, extracted from these crude medicinal herbs.

**Table 2:** Primers used for real-time RT-PCR.

Target Gene	Primer Pairs (5′–3′)	
	Forward	Reverse
*Caspase3*	GAGGCTGACTTCCTGTATGCTT	AACCACGACCCGTCCTTT
*Caspase8*	TTGAACAATGAGATCCCCAAA	CCATTTCTACAAAAATTTCAAGCAG
*Caspase9*	TGCAGTCCCTCCTTCTCAG	GCTTTTTCCGGAGGAAGTTAAA
*CD163*	GGGTCATTCAGAGGCACAGTG	CTGGCTGTCCTGTCAAGGCT
*CD68*	TTGGGAACTACACACGTGGGC	CGGATTTGAATTTGGGCTTG
*Fas*	GCAGACATGCTGTGGATCTGG	TCACAGCCAGGAGAATCGCAG
*FasL*	TCCAGGGTGGGTCTACTTACTAC	CCCTCTTACTTCTCCGTTAGGA
*F4/80*	CTTTGGCTATGGGCTTCCAGTC	GCAAGGAGGACAGAGTTTATCGTG
*Ki67*	GCTGTCCTCAAGACAATCATCA	GGCGTTATCCCAGGAGACT
*MCP-1*	TTCCTCCACCACCATGCAG	CCAGCCGGCAACTGTGA
*p53*	ATGGCTTCCACCTGGGCTTCCTG	CCACAACTGCACAGGGCACGT
*TLR2*	TGCAAGTACGAACTGGACTTCT	CCAGGTAGGTCTTGGTCATT
*TLR4*	GTGCCATCATTATGAGTGCC	CAAGCCAAGAAATATACCATCGAAG
*TNF-α*	TCTTCTCATTCCTGCTTGTGG	TCTGGGCCATAGAACTGATGA
*GAPDH*	TGTGTCCGTCGTGGATCTGA	TTGCTGTTGAAGTCGCAGGAG

## Abbreviations

BSF	Busulfan
TJ7	Hachimijiogan
TJ41	Hochuekkito
TJ107	Goshajinkigan
DMSO	Dimethyl sulfoxide
DW	distilled water
